# m^6^A RNA甲基化在非小细胞肺癌中的研究进展

**DOI:** 10.3779/j.issn.1009-3419.2020.102.35

**Published:** 2020-11-20

**Authors:** 红丽 潘, 雪冰 李, 琛 陈, 亚光 范, 清华 周

**Affiliations:** 1 300052 天津，天津市肺癌转移与肿瘤微环境重点实验室，天津市肺癌研究所，天津医科大学总医院 Tianjin Key Laboratory of Lung Cancer Metastasis and Tumor Microenvironment, Tianjin Lung Cancer Institute, Tianjin Medical University General Hospital, Tianjin 300052, China; 2 610041 成都，四川大学华西医院肺癌中心 Lung Cancer Center, West China Hospital, Sichuan University, Chengdu 610041, China

**Keywords:** 肺肿瘤, m^6^A修饰, RNA甲基化, 表观遗传修饰, Lung neoplasms, N^6^-methyladenosine, RNA methylation, Epigenetic modification

## Abstract

m^6^A修饰是真核生物mRNA中最丰富的修饰之一，该过程受m^6^A甲基转移酶和去甲基化酶的共同调控。m^6^A修饰后的RNA能够被m^6^A识别蛋白特异性识别并结合，进而介导RNA的剪接、成熟、出核、降解和翻译等。目前国内外对于m^6^A修饰及其相关蛋白如何参与非小细胞肺癌发生发展的研究，主要集中于细胞恶性增殖、迁移、侵袭、转移和耐药等方面。m^6^A修饰相关蛋白在肺癌组织标本和血液循环肿瘤细胞（circulating tumor cell, CTC）中表达异常，有望成为肺癌诊断和预后判断的潜在分子标志物。本文围绕m^6^A修饰相关蛋白的组成、作用方式、在非小细胞肺癌恶性进展中的生物学功能，以及针对m^6^A修饰的靶向治疗等方面的研究进展进行综述，旨在为非小细胞肺癌的早期临床诊断和靶向药物的开发提供新思路。

## Abstract (en)

N^6^-methyladenosine is one of the most prevalent mRNA modification in eukaryotes. The regulation of this pervasive mark is a dynamic and reversible process. m^6^A RNA methylation is catalyzed by m^6^A writers, removed by m^6^A erasers and recognized by m^6^A readers, thereby regulating multiple RNA processes including alternative splicing, nuclear export, degradation and translation. Accumulated evidence suggests that m^6^A modification plays a crucial role in the pathogenic mechanism and malignant progression in non-small cell lung cancer (NSCLC), including cell survival, proliferation, migration, invasion, tumor metastasis and drug resistance. Moreover, the expression of m^6^A and its related proteins are dysregulated in clinical samples and circulating tumor cells (CTCs) of lung cancer patients, indicating that m^6^A modification may serve as a novel potential biomarker for the diagnosis and prognosis of lung cancer. In this review, by summarizing a great number of recent reports related to m^6^A's function and its modulators, we aim to provide a new insight on the early diagnosis and drug development in NSCLC therapy.

肺癌是全球范围内发病率和死亡率增长最迅速、对人类健康和生命活动造成最大威胁的恶性肿瘤。全球疾病负担研究报告最新的统计数据^[1]^表明仅2017年全球新增肺癌病例约220万，肺癌死亡病例约190万。中国国家癌症中心公布的数据^[2]^显示2015年我国约有78万例新发肺癌病例，死于肺癌者高达63万人。按照组织类型分类，肺癌主要分为小细胞肺癌和非小细胞肺癌，其中85%的患者为非小细胞肺癌^[3]^。非小细胞肺癌的发生发展是一个多步骤、多因素和多基因参与的极其复杂的过程，所以深入研究非小细胞肺癌的致病机理成为亟待解决的问题。

近年的研究表明，m^6^A修饰在癌症发生发展中发挥着不可替代的作用。m^6^A修饰是指RNA的腺嘌呤（A）第六号（6）氮原子上的单甲基化（m）修饰，简称m^6^A，通常发生在mRNA、lncRNA、circRNA、snRNA、tRNA和rRNA等多种RNA中。研究^[4]^显示，m^6^A修饰的位点具有高度保守性，倾向于出现在共有序列RRm^6^ACH（R=G/A, H=A/C/U）上，这些位点主要分布在RNA的3’UTR、终止密码子或者长外显子附近，对RNA前体的剪接、3’末端的处理、出核转运、降解和翻译等过程起着重要调控作用。本文总结了有关m^6^A修饰的研究成果，阐述了其相关蛋白的组成、作用方式、在非小细胞肺癌恶性进展中的生物学功能以及针对m^6^A修饰的靶向治疗等方面的研究进展，为阐明非小细胞肺癌的发生发展提供新思路，对指导非小细胞肺癌的诊断和治疗提供新靶点。

## 1 m^6^A相关蛋白的组成及功能

m^6^A甲基化修饰最早在酵母tRNA中发现，tRNA发生m^6^A甲基化修饰后有助于维持其三叶草结构的稳定性，提高翻译效率^[5]^。随后发现m^6^A甲基化在mRNA中是最丰富的修饰^[6]^，因此，越来越多的研究关注于mRNA的m^6^A甲基化修饰。m^6^A修饰是一种动态可逆的调节方式，受m^6^A甲基转移酶（m^6^A writers）和去甲基化酶（m^6^A erasers）的共同调控，能够被m^6^A识别蛋白（m^6^A readers）选择性的识别结合，从而转录后调控基因的表达。

### 1.1 m^6^A writers

m^6^A writers，是指m^6^A甲基转移酶复合物，能够催化S-腺苷甲硫氨酸（S-adenosyl methionine，SAM）的甲基转移至腺嘌呤第6位氮原子上。m^6^A writers包括METTL3、METTL14、WTAP、KIAA1429（VIRMA）、RBM15、HAKAI、ZC3H13（KIAA0853）和METTL16等。Knuckles等^[7]^在小鼠胚胎干细胞中过表达了METTL3，期望通过TAP-LC-MS技术筛选鉴定与METTL3相互作用的蛋白。结果表明：在高盐条件（500 mmol/L）下洗脱获得METTL3/METTL14复合物，命名为m^6^A-METTL复合物；在低盐条件（350 mmol/L）下洗脱得到WTAP/KIAA1429/RBM15/ZC3H13/HAKAI复合物，命名为m^6^A-METTL相关蛋白复合物。

#### 1.1.1 m^6^A-METTL复合物

m^6^A-METTL复合物主要包括METTL3和METTL14蛋白。METTL3是最早被发现的m^6^A甲基转移酶复合物成分之一^[8]^，具有甲基转移酶活性。早期通过结构预测和DNA进化分析推测METTL3的功能与甲基转移酶活性相关^[9]^；后期研究^[10]^发现METTL3能够和同源蛋白METTL14形成异源二聚体复合物，而METTL14虽能与RNA结合，但不具备催化活性；最终确定METTL3/METTL14复合物共同催化靶RNA的m^6^A修饰。

#### 1.1.2 m^6^A-METTL相关蛋白复合物

m^6^A-METTL相关蛋白复合物主要是由接头蛋白WTAP、KIAA1429、RBM15、ZC3H13和HAKAI相互结合而形成的复合物。RNA剪接因子WTAP同样不具备甲基转移酶活性，而是作为接头蛋白招募m^6^A-METTL复合物定位到核散斑上，从而影响m^6^A修饰的定位^[11]^。随后的研究发现KIAA1429能够直接与WTAP结合，引导METTL3/METTL14/WTAP复合物结合于靶RNA的3’UTR和终止密码子附近，发生位点选择性甲基化^[12]^。因此，在细胞内敲低KIAA1429的表达后，m^6^A甲基化水平会明显下降^[13]^。后续研究^[12, 14]^证实，接头蛋白RBM15、HAKAI、ZC3H13等能够与WTAP结合，决定METTL3/METTL14/WTAP复合物的正确定位。

#### 1.1.3 METTL16

METTL16是另一具有催化活性的m^6^A甲基转移酶，与METTL3同源，可以结合于U6 snRNA，导致U6第43位的腺嘌呤发生m^6^A甲基化修饰，从而影响U6 snRNP对mRNA前体的剪接^[15]^。METTL16也可以直接与mRNA前体结合，例如，与SAM合成酶MAT2A 3’UTR的一个保守发卡结构结合，在SAM充足或缺乏的情况下，由于停滞时间的差异，导致MAT2A的可变剪接，从而调控细胞内SAM含量的稳态^[16]^。

早期从Hela细胞中分离出的m^6^A甲基转移酶复合物主要包括3个组分，分子量大小分别为30 kDa、200 kDa和875 kDa^[8]^。m^6^A-METTL复合物属于200 kDa组分，但是METTL3/METTL14异源二聚体复合物分子量大小约为120 kDa，远低于200 kDa。m^6^A-METTL相关蛋白复合物属于875 kDa组分，但是WTAP/KIAA1429/RBM15/ZC3H13/HAKAI复合物分子量大小约为600 kDa，远远低于875 kDa。表明仍然有未知的蛋白作为m^6^A甲基转移酶复合物的成员，参与m^6^A甲基化修饰过程，需要进一步深入探讨。

### 1.2 m^6^A erasers

m^6^A erasers，即m^6^A去甲基化酶，能够“擦除”靶RNA的m^6^A甲基化修饰。目前仅发现了两种m^6^A去甲基化酶，包括脂肪质量与肥胖相关蛋白（fat mass and obesity-associated protein, FTO）和alkB同源蛋白5（alkB homolog 5, ALKBH5）。

#### 1.2.1 FTO

FTO是第一个被发现的m^6^A去甲基化酶，在细胞内敲低FTO的表达能够增加mRNA的m^6^A水平，相反，当过表达FTO时，胞内mRNA的m^6^A水平受到抑制。FTO定位于核散斑，这与m^6^A甲基转移酶复合物定位相同^[17]^。m^6^A去甲基化酶FTO的发现，明确了m^6^A修饰是一种动态可逆的调节方式。

然而，FTO作为m^6^A去甲基化酶还存在一些争议：2011年，Jia等^[17]^研究发现FTO的作用底物是m^6^A修饰；2017年，Mauer等^[18]^的研究否认FTO的底物是m^6^A修饰，指出FTO的底物是m^6^Am修饰，从而影响mRNA的稳定性和翻译。在随后的研究中，Su等^[19]^分析了FTO催化m^6^A和m^6^Am修饰的偏好性，发现FTO的偏好性受FTO蛋白定位的影响，在细胞核中FTO催化m^6^A的去甲基化，而在细胞质中的FTO可以同时介导m^6^A和m^6^Am的去甲基化。相比m^6^A修饰，m^6^Am修饰的RNA更加稳定，在细胞内敲低FTO后不影响m^6^Am修饰的RNA的表达，这一发现说明FTO主要通过介导m^6^A修饰的RNA的表达发挥作用。

#### 1.2.2 ALKBH5

ALKBH5是第二个被发现的m^6^A去甲基化酶，属于Fe^2+^和α-酮戊二酸依赖的非血红素加氧酶，能够把m^6^A甲基化位点的N-甲基氧化成羟甲基，从而“擦除”mRNA的m^6^A甲基化。ALKBH5主要定位在核散斑，依赖其去甲基化酶活性影响mRNA的出核转运^[20]^。

目前发现的m^6^A去甲基化酶FTO和ALKBH5均属于AlkB家族，但是二者对m^6^A修饰的擦除作用是相互独立的，互不影响的。FTO和ALKBH5主要定位在细胞核，在细胞质中表达较低，所以细胞质中mRNA的m^6^A修饰是如何被调控的，是否存在定位于细胞质中的m^6^A去甲基化酶，仍需更多研究探索分析。

### 1.3 m^6^A readers

m^6^A readers，即m^6^A识别蛋白，能够选择性识别靶RNA的m^6^A甲基化修饰，进而参与RNA代谢的各个阶段。

#### 1.3.1 YTH家族蛋白

目前m^6^A甲基识别蛋白功能研究比较明确的是YTH结构域家族成员，包括定位于细胞质的YTHDF1、YTHDF2、YTHDF3、YTHDC2和定位于细胞核的YTHDC1，均能通过YTH结构域选择性识别并结合m^6^A修饰位点。YTHDF1与m^6^A修饰位点结合后，招募转录起始因子EIF3A及其复合物，促进翻译的起始和多聚核糖体的形成，提高翻译效率^[21]^；YTHDF2是最早被鉴定的m^6^A识别蛋白之一，识别m^6^A甲基化位点后，进而招募CCR4-NOT脱腺苷酸酶复合体或HRSP12/RNase P/MRP复合物，加速RNA的脱腺苷酸化和切割^[4, 22-24]^。YTHDF3可与YTHDF1协同作用促进m^6^A修饰的mRNA翻译，而YTHDF3与YTHDF2的协同作用会导致m^6^A修饰的靶RNA发生降解^[25]^。YTHDC1与m^6^A修饰位点结合后，能够通过与剪接因子SRSF3和SRSF10的竞争性结合，介导RNA前体的可变剪接及RNA的出核过程^[26, 27]^；而YTHDC2能够提高靶基因的翻译效率和介导RNA的降解^[28, 29]^。

#### 1.3.2 hnRNPs超家族蛋白

与m^6^A修饰相关的hnRNPs超家族蛋白包括HNRNPA2B1、HNRNPC和HNRNPG。RNA结合蛋白HNRNPA2B1可以与核内m^6^A修饰的RNA结合，介导基因的可变剪接。同时，HNRNPA2B1也能够与miRNA初级转录本的m^6^A位点结合，从而招募miRNA微处理复合物蛋白DGCR8，促进miRNA初级转录本进一步的剪接加工^[30]^。随后的研究^[31]^发现，RNA发生m^6^A甲基化修饰后可以引起二级结构的改变，部分m^6^A识别蛋白并非直接识别并结合m^6^A甲基化位点，而是识别构象发生改变的RNA结构，进而调控基因的表达，这一现象被称为m^6^A开关（m^6^A-switch）。通过晶体结构分析和生物信息学预测证实HNRNPA2B1并不含有与m^6^A甲基化位点结合的疏水口袋，可能是采用m^6^A开关机制识别m^6^A修饰位点^[32]^。核糖核蛋白HNRNPC/G对m^6^A修饰的识别结合也是间接性的，通过m^6^A开关机制参与靶mRNA的加工及成熟^[31, 33]^。

#### 1.3.3 其他

真核起始因子EIF3A能够直接和mRNA 5’UTR的m^6^A修饰位点结合，招募43S核糖体复合物，起始帽子结构非依赖型的蛋白翻译^[34]^。IGF2BP1、IGF2BP2和IGF2BP3识别结合m^6^A修饰位点后，增加靶mRNA的稳定性，促进其翻译^[35]^。

总体来说，m^6^A识别蛋白在RNA代谢的各个阶段均发挥了重要作用。

## 2 m^6^A修饰与肺癌

目前，m^6^A修饰在癌症发生发展中的作用及机制研究是肿瘤生物学研究的热点之一。在肺癌的发生发展中，m^6^A修饰发挥着举足轻重的作用。m^6^A相关蛋白表达的异常，能够导致非小细胞肺癌的恶性增殖、迁移、侵袭、转移和耐药等。因此，研究m^6^A修饰在非小细胞肺癌中的生物学功能，鉴定m^6^A修饰的关键因子改变，对阐明非小细胞肺癌发病机制具有重要意义，可以为指导肺癌的临床治疗提供理论依据。

### 2.1 m^6^A修饰与肺癌恶性增殖

肺癌是一种由肺上皮细胞恶性增殖而形成的肿瘤。肿瘤细胞的恶性增殖与细胞的过度分裂、周期紊乱和凋亡调控失常等相关。

研究^[36]^表明m^6^A甲基转移酶METTL3处于甲基转移酶复合物的核心位置，METTL3纯合敲除小鼠是早期胚胎致死的，METTL3条件性敲除鼠表现为体重轻、体积小和发育迟缓等特点，说明METTL3在早期胚胎发育和生存中发挥重要作用。METTL3在肝癌、宫颈癌、乳腺癌和胰腺癌等多种恶性肿瘤中表达均上调，导致SOCS2^[37]^、HBXIP^[38]^和SNAI1^[39]^等mRNA发生甲基化，从而调控肿瘤的恶性进展。在非小细胞肺癌临床样本中，METTL3的表达是明显上调的，并且与肿瘤大小、淋巴结转移和远端转移呈正相关。METTL3能够催化Hippo信号通路的核效应因子YAP发生m^6^A甲基化修饰，促使其翻译，介导非小细胞肺癌的增殖和转移^[40]^。此外，METTL3的第177、211、212和215位赖氨酸残基可以发生SUMO化修饰，显著抑制m^6^A甲基转移酶活性，导致细胞整体基因表达谱的改变，促进非小细胞肺癌的增殖和恶性转化^[41]^。也有报道^[42, 43]^指出，METTL3可以不依赖甲基化酶活性而发挥作用，即直接与表皮生长因子受体（epidermal growth factor receptor, EGFR）和Hippo信号通路的转录调节因子TAZ的m^6^A甲基化位点结合后，招募真核翻译起始因子EIF3H，介导癌蛋白EGFR和TAZ翻译的起始与多聚核糖体的形成，促进肺腺癌细胞的恶性增殖、迁移和侵袭。此外，还有研究^[44, 45]^表明，miR-33a和miR-600可以靶向METTL3而抑制非小细胞肺癌的恶性增殖。

m^6^A识别蛋白YTHDF1在非小细胞肺癌临床样本和细胞株中均高表达。在非小细胞肺癌细胞株中敲低YTHDF1的表达，导致肺癌细胞G_0_期/G_1_期阻滞，细胞周期蛋白CDK2、CDK4和cyclin D1的翻译效率大大降低，显著抑制肺癌细胞的恶性增殖^[46]^。Sheng等^[47]^研究表明YTHDF2在非小细胞肺癌组织中表达上调，通过识别结合磷酸戊糖途径中的关键酶6-PGD 3’UTR的甲基化位点，促进其翻译，加速葡萄糖通过磷酸戊糖途径代谢，为非小细胞肺癌的恶性增殖提供原料。

m^6^A去甲基化酶FTO的表达在非小细胞肺癌组织和细胞系中均上调，它能够“擦除”去泛素化酶USP7 mRNA的m^6^A修饰，增加其稳定性，促进肺癌细胞的恶性增殖^[48]^。而m^6^A去甲基化酶ALKBH5则可以靶向组织金属蛋白酶抑制因子（metallopeptidase inhibitor 3, TIMP3），降低TIMP3 mRNA的稳定性，通过抑制凋亡而促进非小细胞肺癌的增殖^[49]^。USP7 mRNA的m^6^A甲基化修饰被FTO“擦除”后稳定性增加，然而TIMP3 mRNA的m^6^A修饰被“擦除”后稳定性降低，我们推测不同基因m^6^A修饰发生变化后，导致转录后调控方式差异的原因是被不同的m^6^A识别蛋白所选择性识别，从而导致作用方式不同。m^6^A识别蛋白如何选择性识别，具体的分子机制仍需研究者们进一步探索。

### 2.2 m^6^A修饰与肺癌迁移、侵袭和转移

肺癌发展到晚期较易发生转移，这也是导致肺癌死亡率居高不下的重要因素之一，所以m^6^A修饰与肺癌侵袭及转移的关系需要研究者们深入探讨。

在肺腺癌细胞中METTL3依赖m^6^A甲基转移酶活性，导致JUNB mRNA发生m^6^A修饰，增加其稳定性，从而作为转录因子入核开启下游基因的转录，参与转化生长因子-β（transforming growth factor-β, TGF-β）诱导的上皮细胞-间充质转化（epithelial-mesenchymal transition, EMT）的发生，促进肺腺癌细胞的迁移和侵袭^[50]^。在肺癌脑转移过程中，METTL3被报道可以诱导miR-143-3p初级转录本发生m^6^A修饰，加速miR-143-3p的加工成熟，通过靶向血管生成抑制因子VASH1，解除对血管内皮生长因子VEGFA的抑制作用，诱发新生血管的形成，促进肺癌细胞的淋巴结转移和远端转移^[51]^。

Liu等^[52]^研究发现在肺鳞癌中，高表达的m^6^A去甲基化酶FTO通过“擦除”癌基因MZF1 mRNA的m^6^A修饰，增加其稳定性，促进肺鳞癌细胞的恶性增殖、迁移和侵袭。另一m^6^A去甲基化酶ALKBH5在间歇性缺氧处理后的肺腺癌细胞中表达明显上调，进一步研究发现ALKBH5通过“擦除”癌基因*FOXM1* mRNA的m^6^A修饰，促进FOXM1的表达，从而参与肺癌细胞的增殖和侵袭^[53]^。近期的一项研究结果^[54]^表明m^6^A去甲基化酶ALKBH5在非小细胞肺癌中表达明显下调，且其低表达与整体生存期呈负相关；在肺癌细胞中过表达ALKBH5能够明显抑制细胞增殖和迁移，这一结果与前人的研究结果截然相反，推测与细胞培养方式或处理方式的不同相关，具体机制仍需进一步探讨。因此，m^6^A修饰在肿瘤生物学中的调控功能需要更深入、更详细、更全面的研究和探讨。

### 2.3 m^6^A修饰与肺癌耐药性

耐药是导致肿瘤治疗失败的关键因素。研究表明，多药联合可以缓解肿瘤耐药。因此，深入研究耐药的分子机制，可以为指导正确的联合用药和克服耐药提供理论依据。m^6^A修饰相关蛋白在肺癌耐药中也发挥重要的作用。

Shi等^[46]^研究发现在非小细胞肺癌中高表达的YTHDF1能够更好地识别m^6^A修饰的KEAP1 mRNA，促进KEAP1蛋白的表达，导致下游细胞氧化应激反应中的关键因子核因子E2相关因子2（nuclear factor-E2-related factor 2, NRF2）迅速降解，介导耐药基因*AKR1C1*表达的沉默，从而增加了化疗敏感性。m^6^A甲基转移酶METTL3的敲低，也能够提高肺腺癌细胞A549对抗癌药物JQ1的敏感性^[42]^，但是具体机制仍未见报道。

近期Meng等^[55]^深刻剖析了非小细胞肺癌中m^6^A修饰与阿法替尼耐药性的关系：他们以阿法替尼敏感细胞株为对照，发现在阿法替尼耐药细胞株中有更多的基因发生m^6^A修饰，从而导致整体基因表达谱发生了改变；通过基因功能富集分析，发现差异表达的基因主要富集在细胞周期，推测m^6^A修饰的基因扰乱了正常的细胞周期，从而导致非小细胞肺癌产生阿法替尼耐药性。

### 2.4 m^6^A修饰与肺癌诊断及预后

越来越多的研究^[56, 57]^表明，m^6^A修饰可以作为诊断、药物治疗和预后判断的潜在分子标志物。研究人员利用LC-ESI-MS/MS方法检测肺癌单个循环肿瘤细胞（circulating tumor cell, CTC）细胞或CTC细胞簇的m^6^A修饰情况时发现，CTC细胞中的整体m^6^A修饰水平是增加的，这表明从CTC细胞检测m^6^A修饰也许可以成为肺癌的无创诊断方法。未来研究方向会更加关注肺癌早期阶段m^6^A甲基化修饰或m^6^A修饰相关蛋白的表达情况，从而明确m^6^A修饰检测能否作为早期诊断指标。Zhang^[58]^的团队对TCGA数据库509例肺腺癌患者标本的m^6^A相关蛋白的表达进行了分析，发现YTHDF1、YTHDF2和METTL3的表达均明显上调，并且其高表达与总体生存率和无复发生存率呈正相关，他们推测这是通过提高化疗敏感性进而延长了患者的生存期。Liu等^[59]^对13个m^6^A相关蛋白的基因表达进行一致性聚类分析，把肺腺癌患者分为了两组，这两组患者在年龄、种族、肿瘤大小、分期、远端转移方面均有明显差异。通过表达谱分析表明，m^6^A相关基因的表达可以作为肺腺癌晚期患者预后的评判标准。

## 3 针对m^6^A修饰的靶向治疗进展

表观遗传学在恶性肿瘤的发生发展及诊断治疗中具有重要的作用，多个靶向DNA甲基化酶或组蛋白修饰酶的新药已获批用于恶性肿瘤的治疗。DNA甲基转移酶抑制剂Azacitidine和Decitabine已获得美国食品药品监督管理局（Food and Drug Administration, FDA）批准，应用于骨髓增生异常综合征的临床治疗^[60, 61]^。组蛋白去乙酰化酶抑制剂Chidamide已获中国食品药品监督管理局（China Food and Drug Administration, CFDA）批准用于外周T淋巴细胞瘤的治疗^[62]^，目前Chidamide针对非小细胞肺癌的临床实验已进入Ⅲ期阶段。近几年，以m^6^A修饰为核心内容的靶向治疗已经成为药物新靶标的研究热点。主要包括：FTO抑制剂、METTL3-14/WTAP激活剂和联合用药。

### 3.1 FTO抑制剂

m^6^A去甲基化酶FTO是白血病、肺癌、乳腺癌等多种肿瘤发生发展中重要的癌基因之一，在多种肿瘤中普遍高表达，且与预后呈负相关。因此，研究者们致力于筛选或合成FTO抑制剂，为后续抗肿瘤药物的开发奠定了基础。

最早发现的FTO抑制剂是大黄酸，大黄酸是一种天然产物，可以与RNA竞争结合FTO活性位点，从而扰乱了FTO对已发生m^6^A修饰的RNA的去甲基化作用，同时大黄酸也可以抑制去甲基化酶ALKBH2的活性，说明大黄酸并非是特异性靶向FTO的抑制剂^[63]^。研究^[64]^表明用大黄酸处理非小细胞肺癌细胞系PC-9、H460和A549后，能够明显抑制细胞的活性及克隆形成能力，导致凋亡的发生和细胞周期的停滞。小鼠皮下荷瘤实验证实大黄酸能够以时间和剂量依赖的方式抑制肺癌的恶性增殖。之前的研究^[65]^表明大黄酸对小细胞肺癌细胞系GLC4的增殖也有一定的抑制作用，可以诱导细胞发生凋亡。但是由于大黄酸水溶性较差，因此其抗肿瘤效率受到了明显的限制。

随后，研究学者通过高通量荧光偏振检测法筛选抑制FTO或ALKBH5活性的药物，发现非甾体抗炎药MA能够高效的特异性抑制FTO的去甲基化酶活性，并且以浓度依赖方式上调细胞内整体m^6^A修饰水平，而对ALKBH5的表达及活性并未产生影响^[66]^。Demertzi等^[67]^研究发现肺腺癌细胞株A549对MA的敏感性较低，半数抑制率（50% inhibitory concentration, IC_50_）为139 μmol/L，一些MA金属配合物对A549细胞的IC_50_值范围可降至25 μmol/L-40 μmol/L，但是在生物系统的使用浓度仍然过高，所以进一步对MA进行改造，研究发现MA有机锡配合物大大提升了对A549细胞的抗增殖活性，IC_50_值仅为0.43 μmol/L，显著增加了肺腺癌细胞株A549对MA有机锡配合物的敏感性^[68]^。MA配合物是否通过抑制FTO活性来发挥作用，MA配合物对肺鳞癌和大细胞癌的抗癌活性如何呢，有待进一步探讨。

研究者们为了获得特异性和靶向性更强的FTO抑制剂，针对晶体结构进行化合物设计和合成优化，最终筛选到小分子化合物FB23及其羧酸衍生物FB23-2能够特异性靶向FTO。研究^[69]^发现在急性髓系白血病中，羧酸衍生物FB23-2抗增殖活性要优于FB23，能够特异性抑制FTO去甲基化酶活性，显著抑制细胞增殖，促进细胞分化成熟，在一定程度上阻遏了急性髓系白血病的发展。FTO抑制剂FB23及其羧酸衍生物FB23-2在非小细胞肺癌中能否特异性靶向FTO，能否有效抑制肺癌的恶性进展，尚需更多的研究进行验证。

### 3.2 METTL3-14/WTAP激活剂

普遍认为m^6^A修饰的升高可以抑制癌症的恶性进展。前期大量研究聚焦于FTO抑制剂的开发，然而，亦可以通过激活METTL3甲基转移酶的活性来提高m^6^A水平。Selberg等^[70]^基于METTL3/METTL14复合物晶体结构进行了虚拟筛选，发现4个小分子化合物能够与METTL3/METTL14复合物结合并激活甲基转移酶活性，这些小分子化合物能够在细胞水平发挥作用，导致mRNA和rRNA的整体m^6^A水平升高。但是，当使用高浓度（10 μmol/L）小分子化合物处理细胞时，胞内整体m^6^A修饰水平反而降低，我们推测这是细胞为了维持稳态而进行的负反馈调节。在非小细胞肺癌细胞株中METTL3普遍高表达，能够促进非小细胞肺癌的增殖和转移^[42, 43]^。随后Liu等^[59]^分析了大量的肺腺癌和肺鳞癌临床样本中m^6^A相关基因的表达情况，结果表明在肺腺癌和肺鳞癌中METTL3的表达显著上调，并且高表达METTL3的非小细胞肺癌患者表现出较好的预后^[58, 59, 71]^。综合临床数据来看，METTL3在非小细胞肺癌中的高表达能够延长患者的生存期。因此，METTL3-14/WTAP激活剂对非小细胞肺癌的治疗有着充分的理论可行性，相信后续的生物活性实验及临床实验会进一步开展起来。

### 3.3 联合用药

研究^[72]^表明表观遗传修饰的异常能够导致肿瘤对化疗药物的耐受性，所以靶向表观遗传的疗法对逆转耐药提供了可能。Pemetrexed作为晚期非小细胞肺癌的临床药物被广泛使用，但是单药的使用对改善患者的总体生存率具有一定的局限性^[73]^，所以开展了多项针对Pemetrexed的联合用药研究，Bu等^[74]^研究发现FTO抑制剂大黄酸和Pemetrexed联合处理肺腺癌细胞系A549时，IC_50_由2.05 μmol/L降低为0.62 μmol/L，抗肿瘤活性得到明显的提升。Duffy等^[75]^研究发现用阿霉素单药处理肺腺癌细胞株A549时，细胞存活率为65%，而FTO特异性抑制剂MA与阿霉素联用时的细胞存活率仅为16%，这一现象说明MA显著增加了肺腺癌细胞对阿霉素的敏感性。

目前，免疫检验点阻断疗法在抗肿瘤方面取得了较好的成效，已有多个药物获得美国FDA批准应用于肺癌的临床治疗，如Nivolumab、Pembrolizumab和Atezolizumab等。然而，免疫检验点阻断疗法仅对部分肺癌患者有效，并且容易产生耐药性。因此，需要多药联合治疗来扩大应用人群或者克服耐药。近期，Yang等^[76]^研究发现在黑色素瘤细胞中敲低FTO的表达可以增加癌细胞对γ干扰素和抗程序性细胞死亡蛋白1（programmed cell death 1, PD-1）免疫疗法的敏感性。Han等^[77]^研究发现在YTHDF1敲除鼠中皮下注射黑色素瘤细胞，同时联合程序性细胞死亡配体1（programmed cell death-ligand 1, PD-L1）免疫检验点阻断疗法，获得了较好的治疗效果。表明m^6^A相关蛋白在抗肿瘤免疫治疗中发挥着重要的作用。Xu等^[78]^分析了肺鳞癌患者中的免疫细胞类型及m^6^A相关蛋白的表达情况，结果显示滤泡辅助性T细胞的免疫特征可以作为判断肺鳞癌总体生存期的独立预后因素，并且ALKBH5、METTL3、HNRNPC和KIAA1429的低表达能够增加免疫治疗的敏感性。因此，我们有理由相信m^6^A相关蛋白的抑制剂或激活剂与免疫治疗联合用药在非小细胞肺癌治疗中能够提高抗肿瘤效率。

m^6^A相关蛋白的抑制剂或激活剂的开发尚处于起步阶段，现有在研的m^6^A相关蛋白抑制剂或激活剂普遍存在活性低、特异性差、在细胞内影响的表型过于复杂以及其确切的作用机制难以阐明等问题。因此，m^6^A相关蛋白抑制剂或激活剂主要用于临床前实验研究，对癌症患者的治疗效果尚未见报道。随着药物合成与筛选技术的发展、药物改良工艺的进步和多药联合应用的探索，相信未来针对m^6^A修饰的靶向药物一定能够广泛应用于临床抗肿瘤治疗。

## 4 小结与展望

已有研究表明^[79]^，m^6^A修饰的改变在肿瘤发生发展中发挥着重要功能。m^6^A修饰相关蛋白通过调控一系列重要基因的剪接、出核、降解和翻译等，参与多种恶性肿瘤的发生发展。随着m^6^A修饰相关蛋白相继被发现，越来越多的研究集中在m^6^A修饰的潜在机制，从而推动了肿瘤生物学中m^6^A修饰相关信号通路的研究。

当然，关于m^6^A修饰仍有许多未解的谜题：①在生命活动中是否仍存在未知的m^6^A修饰相关蛋白，它们以何种方式参与m^6^A修饰的调控；②迄今为止，已鉴定的m^6^A相关蛋白中m^6^A识别蛋白种类是最多的，每种m^6^A识别蛋白发挥的功能也是不同的，它们是如何选择性识别并结合靶RNA的，它们之间是相互竞争的，还是相互协同的；③m^6^A相关蛋白中有些蛋白具有双重身份，同时具备促癌和抑癌的双重作用，它是以何身份来调控肿瘤的进程，又是以何种作用方式来发挥功能；④目前，已筛选到多种FTO抑制剂及METTL3/METTL14激活剂，研究发现能够明显抑制肿瘤细胞增殖或逆转耐药，但是对癌症患者的治疗效果仍未见报道，所以我们期待更多的临床研究来验证m^6^A相关蛋白是否可以作为恶性肿瘤治疗的新靶点。
